# Cancer-targeted design of bioresponsive prodrug with enhanced cellular uptake to achieve precise cancer therapy

**DOI:** 10.1080/10717544.2018.1477862

**Published:** 2018-06-05

**Authors:** Yuanwei Liang, Wei Huang, Delong Zeng, Xiaoting Huang, Leung Chan, Chaoming Mei, Pengju Feng, Choon-Hong Tan, Tianfeng Chen

**Affiliations:** a The First Affiliated Hospital, and Department of Chemistry, Jinan University, Guangzhou, China;; b Division of Chemistry and Biological Chemistry, Nanyang Technological University, Singapore

**Keywords:** Adverse effects, cancer-targeted, cellular uptake, migration, prodrug

## Abstract

Chemical drug design based on the biochemical characteristics of cancer cells has become an important strategy for discovery of novel anticancer drugs to enhance the cancer targeting effects and biocompatibility, and decrease toxic side effects. Camptothecin (CPT) demonstrated strong anticancer activity in clinical trials but also notorious adverse effects. In this study, we presented a smart targeted delivery system (Biotin-ss-CPT) that consists of cancer-targeted moiety (biotin), a cleavable disulfide linker (S-S bond) and the active drug CPT. Biotin-ss-CPT was found to exhibit potent effects on the migration of cancer cells and induced apoptosis by induction of ROS-mediated mitochondrial dysfunction and perturbation of GSH/GPXs system, as well as activation of caspases. *In vivo* tumor suppression investigation including toxicity evaluation and pathology analysis, accompanied by MR images showed that Biotin-ss-CPT can be recognized specifically and selectively and taken up preferentially by cancers cells, followed by localization and accumulation effectively in tumor site, then released CPT by biological response to achieve high therapeutic effect and remarkably reduced the side effects that free CPT caused, such as liver damage, renal injury, and weight loss to realize precise cancer therapy. Taken together, our results suggest that biotinylation and bioresponsive functionalization of anticancer drugs could be a good way for the discovery of next-generation cancer therapeutics.

## 1. Introduction

Chemical drug design based on the biochemical characteristics of cancer cells has become an important strategy for the discovery of novel anticancer drugs to enhance the cancer targeting effects and biocompatibility, and decrease toxic side effects. Rational design and synthesis of targeted drug delivery system (DDS) is vital and exciting as well as a special form of DDS in which the pharmacologically active payload or medicament only selectively transport to its actual site of action, and not to the non-target tissues or organs (Renoux et al., 2017; Szachniewicz et al., 2017). The DDS has attracted growing research interest over previous decades. It overcomes some of the limitations and barriers of traditional therapy (Mehmood et al., 2016; Dalton et al., 2018; Lin et al., 2018) and symbolizes that the pharmacologically active regimens were preferably selective and effective located at a pre-identified biological target in the vicinity of therapeutic window concentration but its access to non-target normal organs or tissues were limited; thus, toxic effects were minimized while the therapeutic index was maximized (Staben et al., 2016; Zhang et al., 2018). Our previous work has shown the great advantages of cancer-targeted inorganic carrier and nanocarrier platforms, such as excellent bioavailability, large surface area, and controlled release (Liu et al., 2015; Xie et al., 2017; Song et al., 2018; Zhao et al., 2018). CPT, a clinically chemotherapeutic organic drug which inhibits the DNA enzyme topoisomerase I (TOPO I), has demonstrated to be effective against a broad spectrum of solid tumors such as lymphoma, gastric cancer, and colorectal cancer (Chugh et al., 2005; Numbenjapon et al., 2009). Although CPT showed prominent anticancer activity in clinical trials, therapeutic effects are still unsatisfying due to the poor selectivity and the significant side effects (Li et al., 2016; Tian et al., 2017). Due to these disadvantages, the need to improve the selectivity and reduce adverse effects of CPT is very much needed and of greater significance.

Among the active cancer-targeting molecules, biotin has caught a wide attention of many biologists, pharmacists, and medical scientists due to its comparatively small molecular weight, high tumor-specificity, and simple biochemical structure (Liu et al., 2017a; Wu et al., 2018). The binding affinity of avidin to biotin is one of the strongest non-covalent biological interaction (Shetty et al., 2015; Lee et al., 2016a). Due to the overexpression of biotin–receptor uptake systems on the cell surface, biotin or biotin-conjugates can be recognized specifically and selectively by cancers cells, taken up preferentially by cancer cells (Jeon et al., 2014; Liu et al., 2017b). Clinical trials have demonstrated how well using the avidin/biotin system as pre-targeting mechanism works to enhance therapeutic efficacy in different types of cancers (Wang et al., 2005; Lohmueller et al., 2018).

**Figure 1.**
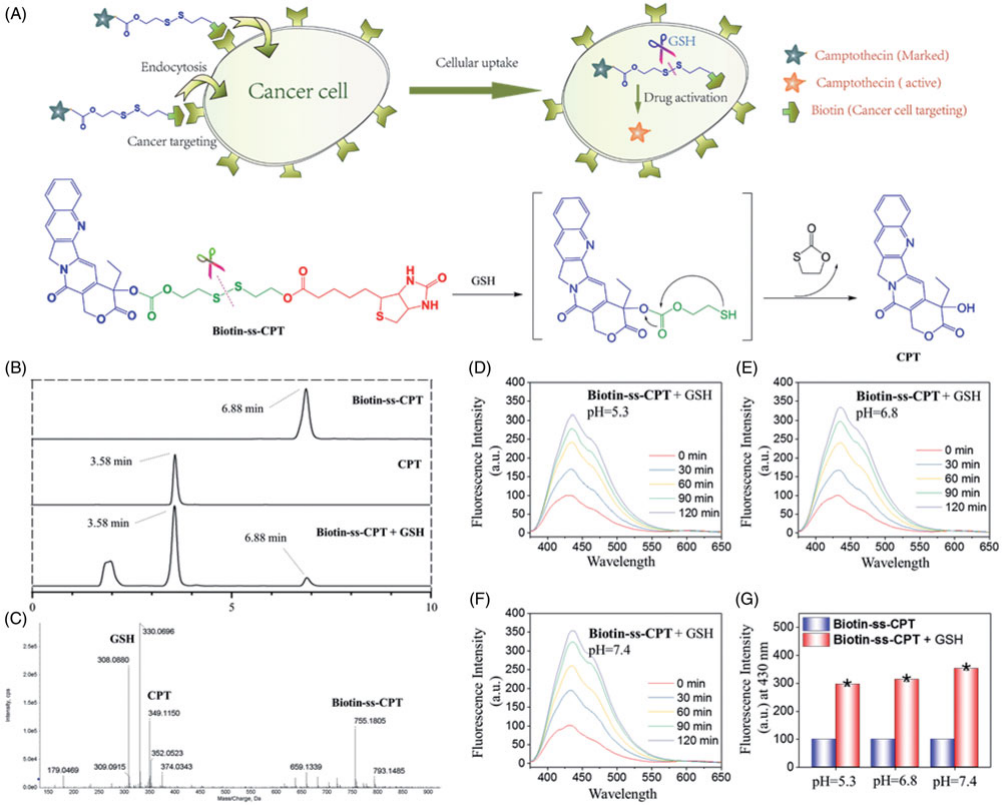
GSH-activatable response of Biotin-ss-CPT. (A) Proposed mechanism in the targeted delivery system (Biotin-ss-CPT) and the CPT activation by GSH. (B) Reverse-phase HPLC chromatograms of Biotin-ss-CPT with GSH. Peaks in the chromatograms were detected by monitoring the UV/Vis absorption at 365 nm. HPLC: Agilent 1260 Infinity II system, Agilent ZORBAX SB C18 (Santa Clara, CA, USA) (250*4.6 mm, 5 µm) Mobile phase A: H_2_O, B: CH_3_CN. 0–20 min: 50–100% B. Flow rate: 1 mL/min. (C) HRMS spectrum of the products from the reaction of Biotin-ss-CPT (20 µM) in a mixed solution of PBS buffer and DMSO (v/v: 4/1) with two equivalent of GSH. Spectrum was obtained 12 h after incubation at 37 °C. The pH dependence at 5.3 (D), 6.8 (E) and 7.4 (F) on the fluorescence spectra variation of Biotin-ss-CPT in the presence of GSH (8 equiv) and the variation in fluorescence intensity at 430 nm (G) before and after incubation with GSH. The data were recorded every 30 min for 2 h after incubation with GSH in mixed solution of PBS buffer and DMSO (v/v: 4/1). Excitation was set at 365 nm.

Disulfides in protein can be used as cellular redox switches via thiol-disulfide exchange which is maintained from micromolecules to macromolecules, with varying degrees of steady-state redox potentials. Recent years, implantation of redox-sensitive disulfide bonds into a drug is an increasingly prevalent method to cause drug release at a target location. A greater number of reports have shown that cellular thiols are indeed served as cellular redox switches involved in the bioreduction processes of redox-sensitive drug (Steiner et al., 2013; Hu et al., 2015; Lee et al., 2016b). Disulfide can be sheared along with the drug delivery system across a poorly reduced extra-cellular space to a strongly reduced intra-cellular microenvironment. Herein, we synthesized a biotin-conjugated prodrug Biotin-ss-CPT and a counterpart Biotin-cc-CPT. The biotin unit in Biotin-ss-CPT can guide the whole system to the tumor zoom, taken up preferentially by cancer cells, reduced by thiol and followed by intramolecular cyclization, resulted in a cleavage of the neighboring organocarbonate bond to liberate CPT (Figure 1(A)). Biotin-ss-CPT was found to exert potent anticancer activity that effectively inhibited the migration, the proliferation and growth by inducing apoptosis on MGC803 cells, while the counterpart Biotin-cc-CPT showed undesirable anticancer activity. Further, mechanism of action on MGC 803 cells was investigated. In addition, the toxicity evaluation, MR-imaging, and pathology analysis of Biotin-cc-CPT, Biotin-ss-CPT, and CPT on MGC803 cancer cells was studied and compared *in vivo*.

## 2. Materials and methods

### 2.1. Materials

Triphosgene, DMAP, EDC, Bis(2,2-diethoxyethyl) Disulfide, hexanediol, and biotin as well as solvent were purchased from Shanghai Civi Chemical Technology Co., Ltd., Shanghai, China. Diamidino-2-phenylindole (DAPI), propidium iodide (PI), and 2′,7′-dichlorofluorescein (H_2_DCF) and Dihydroethidium (DHE) were bought from Sigma Aldrich (St. Louis, MO, USA). Ripa lysing buffer was bought from Thermo Fisher Scientific (Waltham, MA, USA).

### 2.2. Cell lines

MGC803 human gastric carcinoma, GS1 human gastric normal cell, SW620 human caucasian colon adenocarcinoma cells, NCM460 normal human colon mucosal epithelial cells, HepG2 liver hepatocellular carcinoma cells, L02 normal human hepatic cells, MCF-7 breast carcinoma cells, and Hela cervix carcinoma cells were purchased from American Type Culture Collection (ATCC, Manassas, VA, USA). Cells were cultured in DMEM medium containing 10% fetal bovine serum, 100 units per mL of penicillin, and 50 units per mL of streptomycin.

### 2.3. Chemical synthesis

The synthetic route (Scheme S1) and the characterization of (Figures S1–S10) OH-ss-CPT, OH-cc-CPT, Biotin-cc-CPT, and Biotin-ss-CPT were presented in ESI file.

### 2.4. Procedure for the degradation of Biotin-ss-CPT and analysis of drug release by HPLC and HRMS

To a solution of Biotin-ss-CPT in DMSO (0.1 mM, 0.5 ml) GSH in PBS buffer (0.05 mM, 2 mL) was added. The solution was incubated at 37 °C for 12 h then used for HPLC and HRMS analysis. HPLC: Agilent 1260 infinity II system, Agilent ZORBAX SB C18 (Santa Clara, CA, USA) (250*4.6 mm, 5 µm) Mobile phase A: H_2_O, B: CH_3_CN. 0–20 min: 50–100% B. Flow rate: 1 mL/min. Detection wavelength 365 nm.

### 2.5. Stability of Biotin-ss-CPT in water, PBS, human plasma, and culture medium by HPLC and fluorescence analysis

To 3 mL of water, PBS, human plasma, or culture medium, 10 µL of DMSO stock solution of Biotin-ss-CPT was added. The mixture was incubated with gentle shaking for 72 h at 25 °C during which the mixture was measured by HPLC or fluorescence spectra.

### 2.6. Cytotoxicity and antiproliferative activity

Cell proliferation was measured using the MTT assay. Cells were firstly seeded in a 96-well plate (6 × 10^3^ cells/well) and cultured for 20 h. Biotin-cc-CPT, Biotin-ss-CPT, and CPT were added at different final concentrations. 2% culture medium containing 0.1% DMSO was used as a control. After cells were incubated for 72 h, 25 μL of 5 mg/mL MTT PBS solution was added and incubated for 4 h at 37 °C. The supernatant was removed with caution and 150 μL DMSO per well was added to dissolve the formazans. After shaking for 20 min, the absorbance was read at 570 nm on a microplate reader (Bio-Tek, Winooski, VT, USA).

### 2.7. Cell cycle distribution

MGC803 cells were seeded in a 10-cm dish (1 × 10^6^ cells/dish) for 20 h and then exposed to biotin-conjugated CPTs (0.5, 1, 2 μM). After incubation for 48 h, cells were harvested and fixed in 72% ethanol at 4 °C (Yi et al., 2016). After 12 h, the fixed cells were stained with 300 μL DAPI and incubated for 30 min away from light. The cell cycle distribution was determined by flow cytometry (Beckman Gallios, USA). 

### 2.8. Cellular uptake

The cell uptake of drugs was determined by cell lysis assay (Zhou et al., 2016). The cellular uptake of the drugs by the cells was determined by spectroscopy based on the fluorescence spectra of each drug. Briefly, cells were cultured in 10-cm petri dishes at a density of 8 × 10^5^cell/dish for 20 h before treatment. Drugs were added to the dishes at a final concentration of 10 μM for 0, 2, 4, 6, and 8 h, respectively. Then cells were washed by PBS carefully for thrice to remove the extracellular drugs, digested by pancreatin and counted. Cell lysis was carried out by addition of 100 mL of 0.1% Triton X-100. The absorption of each drug was measured by fluorescence microplate reader (CYTATION5, Biotek, Winooski, VT) with the excitation and emission wavelength at 366 nm and 432 nm based on the fluorescence spectra in Figure S11, Figure S12, and Figure S13. The concentration of each drug in cells was calculated in accordance with the standard curve.

### 2.9. Migration assay

Cells were seeded in a 2-cm dish at the concentration of 3 × 10^5^ cells/dish, then scratched the monolayer cells with a 200 μL pipette tip to create wound. Plates were washed with PBS to remove floating cells and debris for three times and then serum-free medium was added, followed by addition of 1 μM free CPT or biotin-conjugated CPTs. Triplicated wells were used for each group. Images for cells migration were photographed at 0 and 24 h. Open wound area was calculated by manual counting.

### 2.10. Mitochondrial observation

Cells were grown on 2-cm glass petri dishes at a density of 8 × 10^4^ cells/dish. After adhering for 20 h, the cells were treated with different concentration of drugs for 24 h at 37 °C. Then cells were washed three times with cold PBS and stained with Mitotracker (red) for 2 h and DAPI (20 μg/mL), the excess Mitotracker and DAPI were washed out with PBS and the cells were observed under a microscope (EVOSs FL Auto Imaging System).

### 2.11. Assessment of mitochondrial membrane potential (ΔΨ m)

Cells cultured in six-well plates and treated with 1 μM biotin-conjugated CPTs, then were trypsinized and resuspended in 500 μL of PBS buffer containing 10 µg/mL JC-1 (Binet et al., 2014). After incubation for 20 min at 37 °C away from the light, the supernatant was removed by centrifugation. The cells were resuspended with PBS and the supernatant was removed again. Finally, cell pellets were suspended in PBS and analyzed by flow cytometry. The percentage of green fluorescence from JC-1 monomers stand for the cells that lost Δ*Ψm*.

### 2.12. Determination of caspase activity

Cells treated with biotin-conjugated CPTs at different concentrations for 48 h were harvested, lysed, and extracted. A BCA assay was used to determine the protein concentration. The cell extracts were incubated with the substrates of caspase-8, -9, and -3 at 37 °C for 2 h. The caspase activity was measured through the fluorescence of the activated caspase with the emission and excitation wavelengths at 380 and 440 nm.

### 2.13. Determination of the intracellular ROS level

Briefly, cells at a density of 2 × 10^5^ cells per mL were incubated with 10 μM DCFH-DA at 37 °C for 30 min. After which the cells were subjected to 1 μM biotin-conjugated CPTs at 37 °C for 2 h in the dark. At intervals of 10 min from 0 min to 60 min, the intracellular ROS level was measured by fluorescence microplate reader (CYTATION5, Biotek) to detect the intensity of DCF fluorescence (excitation and emission wavelength at 488 and 525 nm) (Ma et al., 2018). For DHE (red) probe, the cells were treated as same as DCF and finally subjected to a microscope (EVOSs FL Auto Imaging System: Life Technologies, AMAFD1000 (Thermo Fisher Scientific), Beijing, China) for observation and photographing at intervals of 30 min from 0 to 60 min.

### 2.14. Determination of GSH level

The cell pellet was resuspended in 10 μL of protein removal solution, thoroughly incorporated, centrifuged at 12,000 ×*g* for 10 min. The supernatant was subjected to GSH and GSSG Assay Kit (product No. S0053, Beyotime, Shanghai, China) (Yang et al., 2014) by following the product instructions to determine the GSH level.

### 2.15. Determination of GPXs activity

The activity of GPXs was measured by Total Glutathione Peroxidase Assay Kit (product No. S0056, Beyotime) (Yang et al., 2014).

### 2.16. Biodistribution of biotin-conjugated CPTs

All animal experiments were carried out on the basis of the approval of the Animal Experimentation Ethics Committee of Jinan University. The nude mice were assigned into three groups (*n* = 3 per group) randomly. Equivalent doses of CPT at concentrations of 2 mg/kg (injection volume: 150 μL) were administrated via intravenous injection (caudal vein). The injection saline control was composed of 15% of Cremophor EL +2% of DMSO +83% of normal saline. After 24 h, mice were euthanatized and the heart, liver, spleen, lungs, kidneys, tumor, and blood were collected. The drug concentration in the *ex vivo* organs was quantified by the measurement of drug fluorescence as described in Section 2.8.

### 2.17. Pathology analysis

The main organs including heart, liver, spleen, lungs, kidneys, and tumor were fixed in 4% paraformaldehyde, embedded into paraffin, then stained with hematoxylin and eosin (H&E). The pathological data were captured using a digital light microscope (NIKON, Eclipse Ni-U, Shanghai, China). 

### 2.18. Hematology analysis of MGC803 xenograft nude mice

The blood samples were centrifuged at r/min for 10 min to gain the plasma. Then the plasma was diluted with the same volume of acidified isopropanol (containing 0.75 M HCl solution). The homogenized tissue samples were stored at –20 °C overnight. Being centrifuged at 5000 r/min for 20 min. The supernatant was subjected to blood biochemistry analysis.

### 2.19. Statistical analysis

All experimental values were represented as the mean standard deviation (SD). The data represented at least three independent experiments each done in duplicate. Statistical analysis was performed using the SPSS statistical program (SPSS, Chicago, IL, USA). Significance was established at *p* < .05 (*).

## 3. Results

### 3.1. Rational design and synthesis of cancer-targeted and bioresponsive CPT

The synthetic route for biotin conjugates (Biotin-ss-CPT and Biotin-cc-CPT) is depicted in Scheme S1. Biotin conjugates were prepared by adapting previously established procedures (Liu et al., 2012). CPT was firstly reacted with triphosgene catalyzed by DMAP in the presence of DIPEA. Then the mixture was treated with 2,2′-dithiodiethanol to yield OH-ss-CPT in moderate yield. The finally target prodrug Biotin-ss-CPT was obtained from the esterification reaction of biotin and CPT catalyzed by EDC/DAMP coupling at room temperature. Meanwhile, Biotin-cc-CPT, due to the uncleavable linker (“c − c”), was introduced as a control system.

### 3.2. GSH-activatable response and the physiological stability of biotin-ss-CPT

The anticipated CPT release was confirmed by reverse-phase HPLC and HRMS analyses (Figure 1). Free Biotin-ss-CPT was eluted at 6.88 min in HPLC chromatogram (Figure 1(B)) and corresponding to the HRMS spectrum (Figure S3) peak was at 755.1862 m/z [M + H]^+^. As GSH was added to Biotin-ss-CPT, Biotin-ss-CPT is sheared by GSH. As chemical reaction involved, this scission was subjected to intramolecular cyclization, followed by ring-forming condensation, resulting in the liberation of CPT. The peak at 6.88 min, paralleling to Biotin-ss-CPT, decreases a lot and new strong fragments peaks appear that elute at 3.58 min, well matched with free CPT, paralleling to the peak intensity at 349.1150 m/z [M + H]^+^ in HRMS spectral analysis (Figure 1(C)). Together, the disulfide bond present in Biotin-ss-CPT was demonstrated to be cleaved by GSH.

In consideration of the tumor microenvironment, the pH dependence (5.3, 6.8, and 7.4) on the GSH-induced fluorescence changes of the prodrug were studied. Results (Figure 1(D), 1(E), and 1(F)) showed that different pH value all induced apparent enhancement in fluorescence intensity. And the increased amplitude among pH 5.3, 6.8, and 7.4 was slightly different (7.4 > 6.8 > 5.3) which can be seen in Figure 1(G). These data demonstrated that pH at 5.3, 6.8, and 7.4 all resulted in the liberation of CPT from the prodrug.

Furthermore, CPT release from the prodrug at different GSH concentrations was also investigated by HPLC analysis and fluorescence spectra. This was done by treating Biotin-ss-CPT (20 μM) with 0 − 8 equivalent of GSH then the variation was monitored by the peak area and fluorescence intensity, respectively. Figure S14(A) showed that with the increased concentration of GSH reacting with Biotin-ss-CPT in a mixed solution of PBS buffer and DMSO (v/v:4/1) for 2 h, the peak area of CPT released from Biotin-ss-CPT increased correspondingly and meanwhile the peak area of Biotin-ss-CPT decreased. The GSH response to Biotin-ss-CPT was also further confirmed by the fluorescence spectra that the fluorescence intensity raised (Figure S14(B)) gradually accompanied by the increased concentration of GSH (0–8 equivalent). However, treating GSH with the control group Biotin-cc-CPT did not experience any change in the fluorescence intensity (Figure S14(C)). This well demonstrated the concentration dependence of GSH to the release of CPT from Biotin-ss-CPT and nonresponse of GSH to Biotin-cc-CPT.

To evaluate the competition from other potential analytes, the interaction of Biotin-ss-CPT with other thiols such as Homocysteine (Hcy) and Cysteine (Cys), and various thiol-free amino acids were investigated. By adding Hcy or Cys to Biotin-ss-CPT, variation in the fluorescence intensity experienced a similar increase (Figure S15(A) and S15(B)) which are close to the case of GSH. However, no apparent variation in fluorescence was witnessed when exposing to various thiol-free amino acids (Figure S15(C)). The results came to a conclusion that the prodrug would experience thiol-cleavage in the biotic environment but not decompose in the presence of other potential interferences such as various amino acids.

To confirm that the prodrug Biotin-ss-CPT did not decompose under physiological conditions, we also investigated the drug stability in water, PBS, human plasma, and culture medium by fluorescence spectrum and HPLC analysis at intervals. The results show that the fluorescence spectrum did not witness variations in water, PBS, human plasma, and culture medium during the 72-h incubation (Figure S16A), Simultaneously, HPLC analysis results showed that the peak area and peak height of Biotin-ss-CPT nearly remained unchanged (Figure S16(B)), demonstrating that Biotin-ss-CPT existed stably under physiological conditions.

In the light of these data, Biotin-ss-CPT existed physiologically and was able to be cleaved by GSH, resulting in the liberation of CPT.

### 3.3. Enhancement of cancer selectivity

Given that Biotin-ss-CPT can be activated intracellularly to liberate CPT, its performance on cell lines was firstly investigated *in vitro*. The cytotoxicity of free CPT, Biotin-cc-CPT, and Biotin-ss-CPT against a few tumor cells and their corresponding normal cells was evaluated using MTT assays (Figure S17). The IC_50_ value of antiproliferative activity is calculated and displayed in Table 1.

**Table 1.** *In vitro* cytotoxic activity IC_50_ (μM) of Biotin-cc-CPT, Biotin-ss-CPT, and CPT.

	IC_50_ (μM)		
Complex	Biotin-cc-CPT	Biotin-ss-CPT	CPT
MGC803	24.0	0.92	0.74
GS1*	31.8	4.5	0.79
SW620	19.3	1.2	0.69
NCM460*	29.2	5.3	0.81
HepG2	21.9	1.5	0.89
L02*	29.5	5.8	0.93
MCF7	16.2	0.93	0.53
Hela	15.9	0.61	0.36
Si^a^	1.33	4.89	1.07
Si^b^	1.51	4.40	1.18
SI^c^	1.35	3.86	1.05

*Human normal cells. Cells were treated for 72 h.

^a^Safety Index = IC_50_(GS1)/IC_50_(MGC803).

^b^Safety Index = IC_50_ (NCM460)/IC_50_ (SW620).

^c^Safety Index = IC_50_ (L02)/IC_50_ (HepG2).

Specifically, Biotin-ss-CPT exerted potent antiproliferative activity on MGC803 (human gastric carcinoma cell line), MCF7 and hela cell lines with IC_50_ of 0.92, 0.93, and 0.61 μM, respectively, which was close to CPT with IC_50_ of 0.74, 0.53, and 0.36 μM, respectively. For other two tumor cell lines, SW620 and HepG2, Biotin-ss-CPT still showed good antiproliferative activity, with IC_50_ value of 1.2 and 1.5 μM, respectively, only about 1.5-fold of CPT (0.69 and 0.89 μM, respectively), demonstrating that the anticancer activity of Biotin-ss-CPT was close to the parent drug CPT.

Biotin-cc-CPT, the counterpart of Biotin-ss-CPT, exerted more than 10-fold poorer activity against above cell lines (with IC_50_ value of up to 24.0, 19.3, and 21.9 μM to MGC803, SW620, and HepG2 cell lines, respectively) compared with Biotin-ss-CPT. This was mainly because of the release of CPT from Biotin-ss-CPT through the cleavage of the disulfide linkage, while Biotin-cc-CPT was completely unable to liberate CPT in cancer cells, resulting in poor activity. However, it was worth noting that the parent drug CPT still exerted an apparent cytotoxicity toward noncancerous cells GS1, NCM460, and L02 (the counterpart of MGC803, SW620, and HepG2), while Biotin-ss-CPT displayed much lower cytotoxicity comparing with CPT. Meanwhile, the safety index of Biotin-ss-CPT on MGC803, SW602, and HepG2 cell lines were 4.89, 4.40, and 3.86, respectively, which were apparently higher than that of CPT (1.07, 1.18, and 1.05, respectively). These data pointed to a fairly high selectivity of prodrug Biotin-ss-CPT Based on the potent activity of Biotin-ss-CPT on MGC 803 cells, and the good selectivity between MGC803 and GS1 cell lines, as well as the limited reports about the biotin-conjugated CPTs on MGC803 cell lines. MGC 803 cells were used as cell model *in vitro* and in *vivo* in this study.

### 3.4. Enhancement of cellular uptake and anti-migration effects

**Figure 2.**
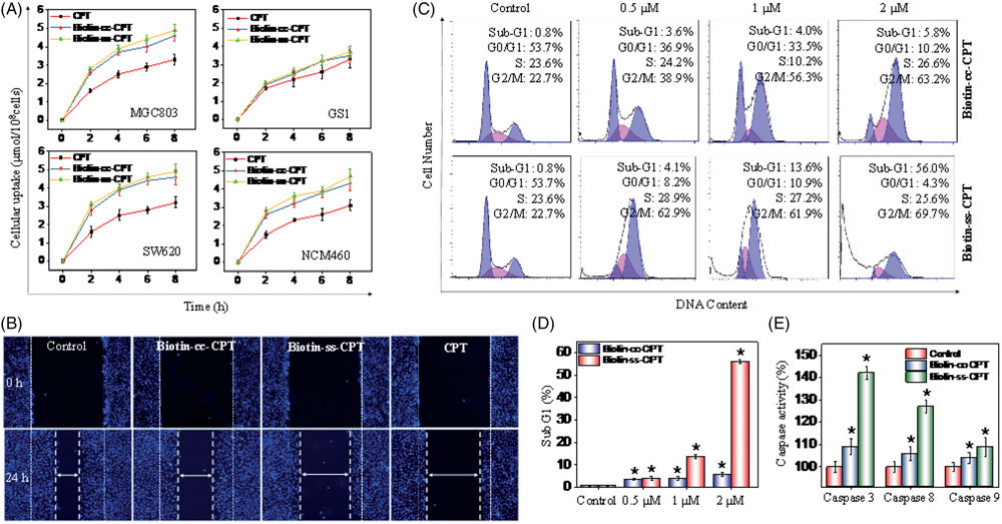
(A) Intracellular uptake of CPT and biotin-conjugates on tumor cells (MGC 803 and SW620) and corresponding normal cells (GS1 and NMC460) during of 8-h period. Error bars represent SD of *n* = 3 data sets. (B) Inhibitory effects of migration of Biotin-ss-CPT, Biotin-cc-CPT and free CPT on MGC 803 cell lines. Cells were treated with 1 μM biotin-conjugated CPTs or free CPT for 24 h then Nuclei (blue) were stained with DAPI. (C) Flow cytometric analysis of MGC 803 cell cycle distribution. (D) The proportional (%) of Sub-G1 phase population. (E) The activity of caspase 8, 9 and 3 on MGC 803 cells after treatment with biotin-conjugated CPTs. MGC803 cells were treated with CPTs at 0.5, 1, and 2 μM for 48 h.

A variety of drug delivery systems have been exploited for the purpose of improving drug delivery and cellular uptake. The high affinity in biotin-avidin has paved the way for many applications, such as biochemistry, biomedicine, and pharmacochemistry (Schmidt & Healy, 2013; Anabuki et al., 2018). Based on the particularly tight interaction between biotin and receptor, we studied and compared the cellular uptake of biotin-conjugated CPTs and free CPT on tumor cells MGC 803 and SW620, and their counterpart normal cells. As we can see from Figure 2(A), the cellular uptake of biotin-conjugated CPTs (Biotin-cc-CPT and Biotin-ss-CPT) on tumor cells (MGC 803 and SW620) both obviously maintained at higher level than free CPT after incubation for 8 h, while the rate of biotin-conjugated CPTs entering into normal cells (GS1 and NMC460) were slow down as a whole compared with tumor cells, which are not so much as CPT. Besides, the prodrug Biotin-ss-CPT gain slightly higher rate of cellular uptake than Biotin-cc-CPT. This faster intracellular uptake presumably was due to the excellent biological responsiveness of Biotin-ss-CPT.

In most instances, tumor metastasis causes higher death rate than the primary tumor dose, and it has become a major cause of death in cancer treatment (Liang et al., 2016). Therefore, the suppression or prevention of metastatic diseases becomes especially important in the therapeutic process. The migration inhibitory effects of Biotin-conjugated CPT and free CPT was investigated on MGC803 cells. As shown in Figure 2(B), the untreated cells (control) nearly moved and repopulated to a majority of the scratched wound region in 24 h. Besides, Biotin-cc-CPT (1 μM) treated cells also showed aggressive repopulation tendency, spread out for a larger part, accounting for about 75% migrated cells (Figure S18) compared with control. Nevertheless, the prodrug Biotin-ss-CPT (1 μM), which releases CPT in cells, effectively retarded the migration of MGC 803 cells that only limited migrated cells can be seen in the scratched wound region in 24 h. Cells treated with Biotin-ss-CPT presented only 22% migrated cells in comparison with control, approached to that of free CPT, only 18% cells migrated. In the light of these data, Biotin-ss-CPT present fairly great potential in decreasing the risk of migration of MGC 803 cells, whereas Biotin-cc-CPT, without biological responses to liberate CPT, show poor suppression effect of metastasis on MGC 803 cells. These results suggest that Biotin-ss-CPT greatly improved cellular uptake comparing with CPT and exerted potent anti-migration effects on MGC803 cells.

Meanwhile, the targeting ability of the prodrug that binds to the receptor was performed by a competitive binding assay of biotin. MGC 803 cells were pre-incubated with different concentration of free biotin (0, 1, 5, 10, and 20 µM), and then exposed to free CPT (5 µM) or Biotin-ss-CPT (5 µM) for 72 h. The results show that, as the concentration of biotin increased from 0 to 20 µM, the cell viability of CPT group showed no obvious change, merely from 28 to 35% (Figure S19(A)). However, this situation was totally different for Biotin-ss-CPT. The cell viability of Biotin-ss-CPT without biotin pretreatment was only 31% (Figure S19(B)). When the cells were pre-incubated with biotin and as the concentration rose, the cell viability increased apparently. As the concentration increased to 20 µM, the cell viability increased up to 61%, which was significantly higher than that of CPT (35%).

This targeting ability was also confirmed by the fluorescence intensity of the drugs. The MGC 803 cells were pre-incubated with biotin (0 or 20 µM) for 2 h, then exposed to Biotin-ss-CPT (5 µM) or free CPT (5 µM). The results (Figure S19(C)) show that the fluorescence intensity of CPT showed no difference with or without pre-incubation of biotin. On the contrary, Biotin-ss-CPT with biotin pre-incubation for 2 h in advance presented much lower fluorescence intensity, demonstrating that the cellular uptake of Biotin-ss-CPT is reduced by pre-incubation of biotin. The results were well in line with the results of cell viability. Taken together, these results confirm the important role of biotin in the cancer-targeted delivery of CPT.

### 3.5. Induction of cell cycle apoptosis and caspases activity

Cell cycle anomalies are often observed after different types of cell injury. Mitotic arrest (checkpoints) and apoptosis are two major events that regulate cell growth (Liang et al., 2014). To investigate the effects of the synthesized agents on cell cycle, flow cytometric analysis was performed. As shown in Figure 2(C), Biotin-cc-CPT-treated cells caused unremitting increase in the G2/M percentage (38.9 to 72.9%) in a dose-dependent manner (0.5 to 2 μM) compared with control, whereas no significant change in the percentage of Sub-G1 phase was witnessed, revealing that Biotin-cc-CPT mainly induced cell cycle G2/M-phase arrest. On the other hand, when cells were exposed to Biotin-ss-CPT, a gradually upward trend in Sub-G1 phase was found in a dose-dependent manner. The percentage of Sub-G1population was found to increase noticeably (from 4.1 to 56.0%) compared with control (0.8%). Notably, the Sub-G1 population gap between Biotin-ss-CPT and Biotin-cc-CPT treated cells is rather large in the concentration of 1 μM and 2 μM, as reflected in Figure 2(D).

Caspases are vital drivers of apoptotic cell death. Cells that fail to properly modulate caspase activity can result in untimely cellular apoptosis. Our results show that Biotin-ss-CPT effectively activated apoptotic initiators caspase-8 (extrinsic mediated) and caspase-9 (intrinsic mediated), which subsequently induced the activation of executive caspase-3 and resulted in apoptosis (Figure 2(E)). Nevertheless, the activation of caspase 3/8/9 by Biotin-cc-CPT was rather limited. In the light of these results, Biotin-cc-CPT affected the replication of DNA and caused cell cycle arrest in G2/M-phase, while Biotin-ss-CPT prevented the cell proliferation and growth by causing increased cells entering into Sub-G1 phase and activation of caspase activity, resulting in cell apoptosis.

### 3.6. Induction of ROS-mediated mitochondrial dysfunction and perturbation of GSH/GPXs system

**Figure 3.**
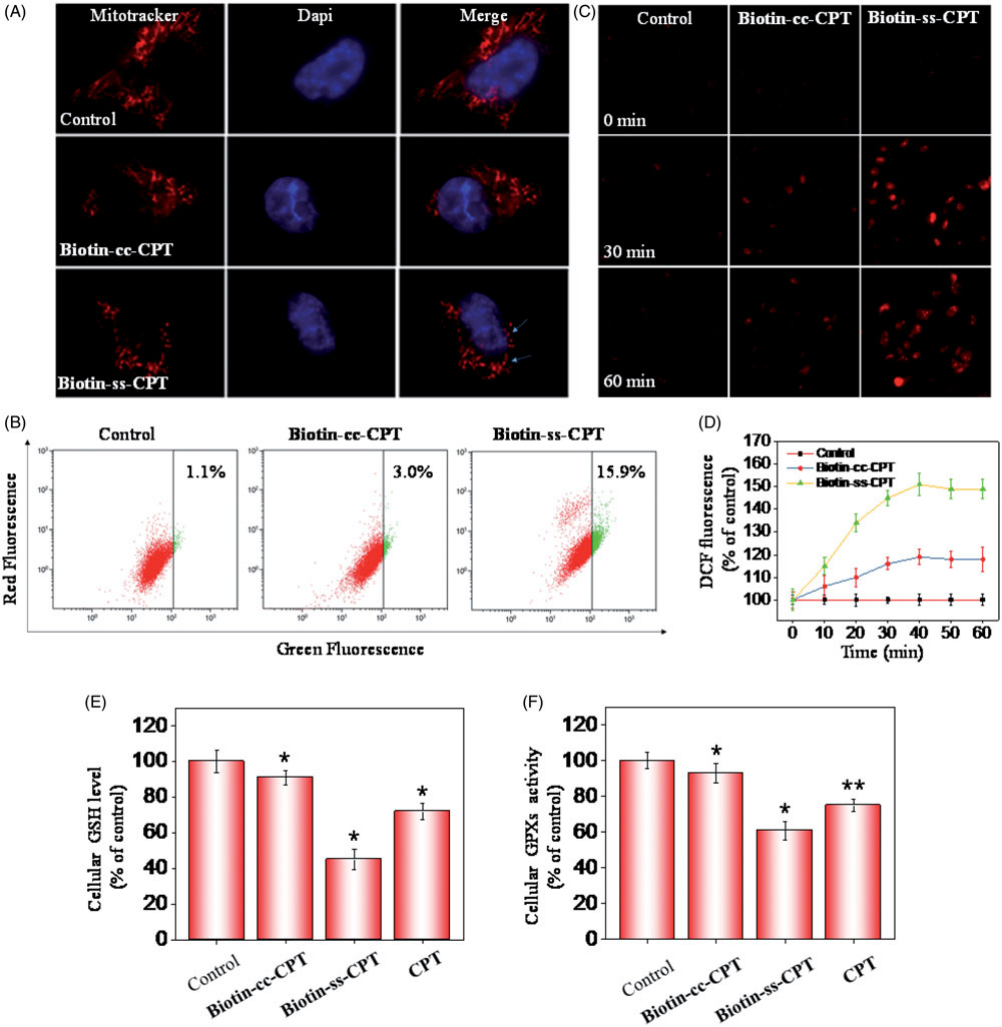
Induction of ROS-mediated mitochondrial dysfunction and perturbation of GSH/GPXs system. (A) Photomicrographs of mitochondria fission and cytoplasmic shrinkage induced by 1 μM biotin-conjugated CPTs as detected using Mitotracker & DAPI co-staining. The state of mitochondrial fission is indicated by the arrows. (B) Flow cytometric analysis of the changes in Δ*Ψm* on MGC 803 cells treated with Biotin-cc-CPT or Biotin-ss-CPT. The proportion in the right region emitting green fluorescence stands for the percentage of cells that due to the loss of Δ*Ψm*. (C) ROS generation triggered by biotin-conjugated CPTs on MGC 803 cells. Cells incubated with 10 μM DEH in PBS for 30 min were exposed to biotin-conjugated CPTs. (D) ROS generation marked by 10 μM DCF fluorescence. Cells treated with 1 μM of biotin-conjugated CPTs. (E) and (F) Changes in intracellular GSH level and GPXs activity. Cells were treated with 1 μM of biotin-conjugated CPTs. .01 < *p* ≤ .05 and *p* ≤ .01 are considered to be statistically significant and highly significant and are denoted as “*” and “**”, respectively. Student’s t test.

In many cell types, mitochondria spreads throughout the whole cytoplasm, the structures of which tend to be long, tubular, or branched (Westermann, 2010). As shown in Figure 3(A), treating cells with Biotin-ss-CPT obviously gave rise to mitochondrial fission (arrows denoted) and cytoplasmic shrinkage. Whereas no apparent change in mitochondrial morphology were seen in Biotin-cc-CPT-treated cells. These results demonstrated the activation of mitochondrial-mediated apoptosis caused by Biotin-ss-CPT. Mitochondrial dysfunction has been displayed to participate in the mechanism of inducing apoptosis and is vitally important to the apoptotic pathway (Westermann, 2010). In fact, the opening of the mitochondrial permeability transition pore would result in trigger depolarization of the transmembrane potential (deltapsi) and release of apoptogenic factors. In some apoptotic mechanism, loss of deltapsi tend to be an early event in the apoptotic process (Chamberlain et al., 2013). To evaluate the role of mitochondria in prodrug-induced apoptosis, the status of Δ*Ψm* was investigated on Biotin-cc-CPT and Biotin-ss-CPT-treated MGC 803 cells by JC-1 flow cytometric analysis. As shown in Figure 3(B), no apparent effect in the depletion of Δ*Ψm* was found after treating MGC 803 cells with Biotin-cc-CPT (1 μM), as reflected by the limited fluorescence shift from red to green. The proportion of depolarized mitochondria on MGC 803 cells only increased from 1.1% (control) to 3.0%. Nevertheless, significant fluorescence shift from red to green was witnessed exposing cells to Biotin-ss-CPT (1 μM), the proportion of depolarized mitochondria on MGC 803 cells increased to 15.9%, which demonstrate Biotin-ss-CPT gave rise to a rapid dissipation of Δ*Ψm*. These data showed that alterations in Δ*Ψm* made contribution to Biotin-ss-CPT induced apoptosis on MGC 803 cells.

Reactive oxygen species (ROS) is a collective term that contains a variety of oxygen-containing, reactive, and short-lived molecules, such as peroxides (H_2_O_2_), superoxide (O_2_^−^), and hydroxyl radical (OH•). There are many evidences showing that ROS act as a critical mediator in cell apoptosis in the biological context (Martin et al., 2014). Cells are particularly sensitive to ROS-resulted redox imbalance that a variety of biological responses were subsequently activated, such as cell arrest, initiation of signal transduction pathways, and repair of damaged DNA. Which in all probability determine whether cells survive or not ultimately (Chung et al., 2015). In this study, to investigate ROS generation triggered by Biotin-ss-CPT and Biotin-CC-CPT on MGC 803 cells, the probes: 2′,7′-dichlorofluorescein (H_2_DCF) and dihydroethidium (DHE) were used. DCF is especially sensitive to hydrogen peroxide (H_2_O_2_), while DHE to superoxide (O_2_^-^). The reduced forms of H_2_DCF is non-fluorescent, H_2_DCF forms a green fluorescence product (DCF) once the acetate groups are removed by intracellular esterase and oxidized (Guo et al., 2014). On the other hand, by reacting with superoxide anions, DHE forms a red fluorescent product (2-hydroxyethidium). As shown in Figure 3(C), after incubation with Biotin-cc-CPT on MGC 803 cells, the DHE probe turn a bit brighter in fluorescence color after 30 min comparing with control, but the brightness did not see any obvious change after the next 30 min. Still, exposing cells to Biotin-ss-CPT resulted in apparent fluorescence change. The red color turn much brighter along with time (30 and 60 min) after incubation with Biotin-ss-CPT, demonstrating that Biotin-ss-CPT showed a dose-dependent increase in intracellular ROS. Besides, microplate reader showed the curvilinear trend rose dramatically in the first 40 min period for probe H_2_DCF when cells were exposed to Biotin-ss-CPT (Figure 3(D)), and then a peak at the period of 30 to 40 min. Nevertheless, Biotin-cc-CPT-treated cells only witnessed slight ascend and peak at about 40 min. These results point to the important role of ROS in Biotin-ss-CPT-induced cell apoptosis.

The GSH-GPXs system plays a crucial role in a variety of biochemical processes including diverse biological oxidation-reduction reactions, transport and protection against xenobiotics (Shimodaira et al., 2017). Many literature reports have shown that GSH and GPXs are known as key targets for apoptosis regulators (Wang et al., 2017; Zhao et al., 2017). The synthetic prodrug Biotin-ss-CPT can lead to the depletion of GSH and the release of CPT. This may subsequently give rise to the inactivation of GPXs, by taking this feature into consideration, as well as the results that Biotin-ss-CPT-induced change in mitochondrial morphology, loss of Δ*Ψ m* and induction of intracellular ROS generation, GPXs were supposed to play an important role in Biotin-ss-CPT-induced apoptosis pathway. Thereupon, MGC803 cells were collected after treated with Biotin-cc-CPT, Biotin-ss-CPT, and CPT to detect changes in GSH level and GPXs level. Results show that Biotin-ss-CPT and CPT dramatically decreased in both GSH levels and GPXs activity (Figure 3(E) and 3(F)). Whereas Biotin-cc-CPT hardly induced changes in GSH levels and GPXs activity. Notably, Biotin-ss-CPT caused more severe decline in GSH levels and GPXs activity than CPT did, suggesting that depletion of thiol by Biotin-ss-CPT made a contribution. Taken together, the induction of ROS-mediated mitochondrial dysfunction and perturbation of GSH/GPXs system involved in Biotin-ss-CPT-induced apoptosis mechanism.

### *3.7.* *In vivo* anticancer activity

**Figure 4.**
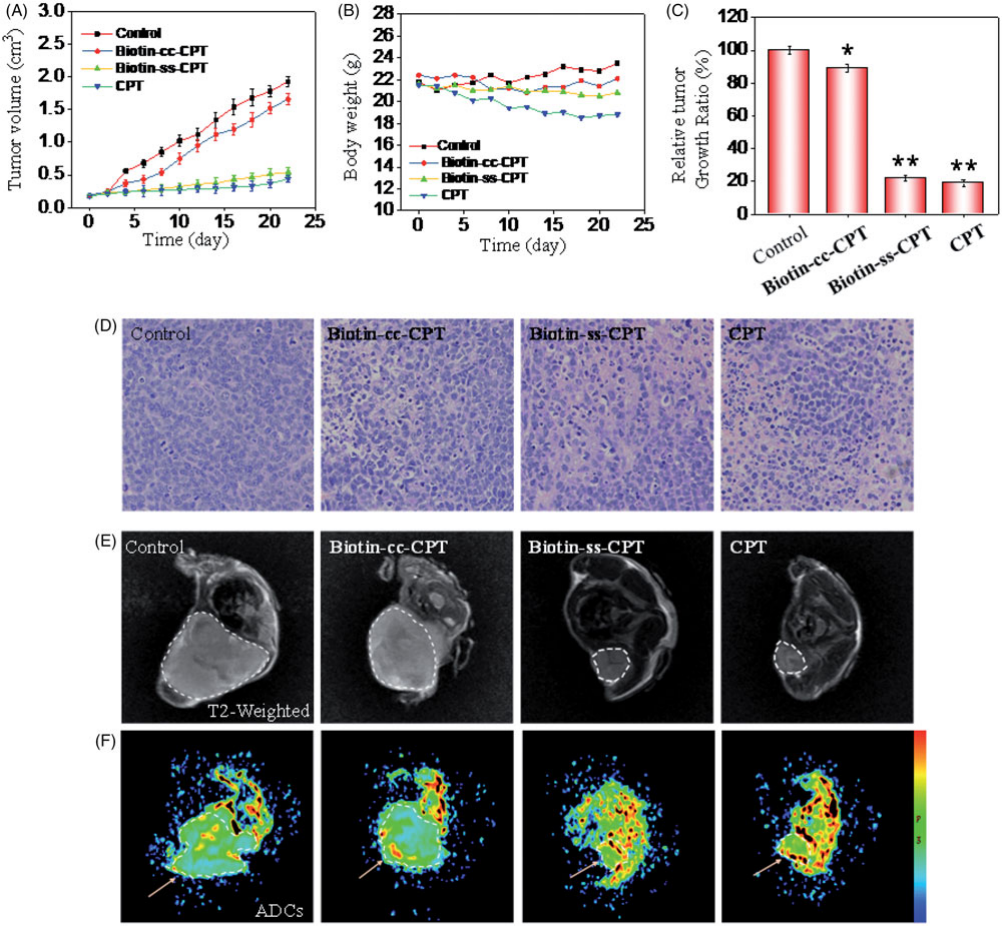
*In vivo* anticancer activity. (A) Changes in tumor volume of different drug-treated MGC803 cancer nude mice for 22 days. (B) Body weight of xenograft MGC 803 cancer nude mice treated by different drugs. (C) Relative tumor growth ratio. (D) H & E stained tissue sections from tumor of the mouse treatment of different drugs. (E) T2 MR images of tumors treated with different drugs after 22-day drug injection. (F) Pseudo-color images of the ADCs.

**Figure 5.**
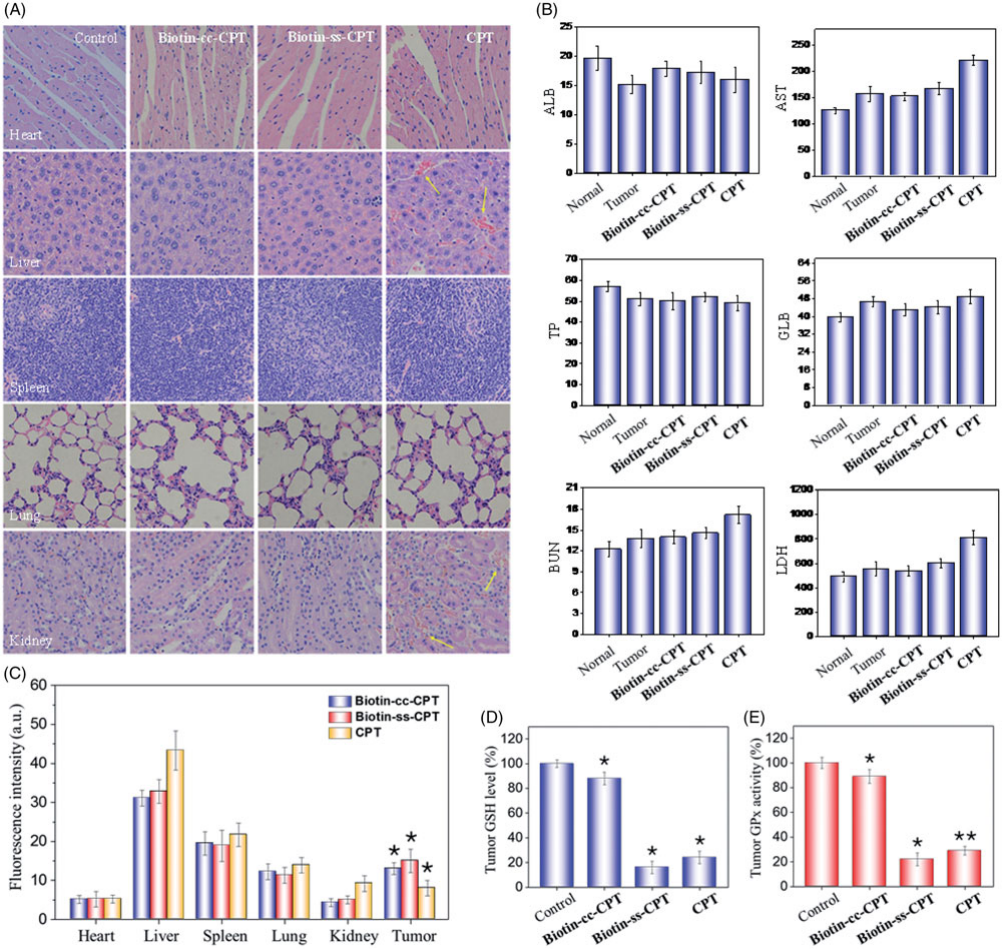
(A) H & E staining section of heart, liver, spleen, lung, kidney from nude mice after treatment with equivalent dose of 2 mg/kg CPT after 22 days treatment. (B) Blood biochemistry analysis of the indices of ALB, AST, TP, GLB, BUN, and LDH in mice. (C) *In vivo* biodistribution of different drugs in major organs. (D) Tumor GSH level. (E) Tumor GPx activity. *p < .05, **p < .01. Student’s t test.

Investigations were also carried out to examine the therapeutic efficacy of biotin-conjugated CPTs in MGC 803 tumor-bearing nude mice. The nude mouse bearing the tumors was treated with biotin-conjugated CPTs through tail intravenous injection every other day at a CPT equivalent dose of 2 mg/kg. As shown in Figure 4(A) and 4(B), Biotin-cc-CPT hardly inhibited the growth of the tumor, as confirmed by the sustained growth in tumor volume which were side by side with and close to the control group. However, which worth the whistle was that Biotin-ss-CPT effectively suppressed tumor growth that nearly approached to free CPT. The relative tumor growth ratio of Biotin-ss-CPT and free CPT were 22 and 18%, respectively, whereas up to 90% is Biotin-cc-CPT (Figure 4(C)). The data symbolized that the prodrug Biotin-ss-CPT significantly inhibited the proliferation of MGC 803 tumor *in vivo*. In most cases, drug toxicity is likely to bring about variation in the body weight. Figure 5(B) shows the change in body weight of different drug-treated mice. Control group saw a slight increase in the body weight in the 22-day period. Biotin-cc-CPT and Biotin-ss-CPT-treated mice basically remained unchanged. However, free CPT caused apparent weight loss. From the rate of body weight change, the control group was 107%, while Biotin-cc-CPT and Biotin-ss-CPT were 101 and 97%, respectively. The CPT alone was 86% (Figure S20), demonstrating that toxicity of free CPT bring obvious weight loss. While Biotin-ss-CPT, which had nearly the same activity with CPT, did not lead to weight loss. Furthermore, Biotin-ss-CPT and CPT both induced more remarkable tumor destruction than Biotin-cc-CPT which can be observed by H&E analysis (Figure 4(D)), indicating the outstanding tumoricidal efficacy of Biotin-ss-CPT.

In addition, MR including T2 weight images and pseudo-color images of the ADCs (Slow Diffusion Components) were also employed to confirm the anticancer activity of *in vivo* from the view of diagnostic imaging. As shown in Figure 4(E), compared with control and Biotin-cc-CPT groups, Biotin-ss-CPT and CPT groups effectively inhibited the tumor growth that the tumor area were obviously smaller than that of control and Biotin-cc-CPT groups in T2 weighted image and ADCs. Moreover, we found that there was a great gap between the ADCs value that reflects the activity and density of cells. As shown in Figure S21, ADCs value of Biotin-ss-CPT and CPT group distinctly increased to 8.3 × 10^−4^ and 8.8 × 10^−4^ compared with the control group (4.5 × 10^−4^), indicating the potent ability of Biotin-ss-CPT in reducing cancer cell activity and density. These results demonstrated Biotin-ss-CPT exerted outstanding tumoricidal efficacy *in vivo*.

### 3.8. Biotinylation decreases *in vivo* toxicity of CPT

*In vivo* anticancer activity above showed that free CPT bring obvious weight loss. While Biotin-ss-CPT did not. This, to a certain extent, had reflected the lower toxicity of Biotin-ss-CPT in comparison with CPT. Potential drugs mostly show high efficiency in tumor tissues and low toxicity in non-tumor ones, which could reduce side effects during chemotherapy. Still, numerous blockbuster drugs on sale are hard to break this balance that high efficiency is likely to be accompanied by high toxicity and side effects. Therefore, we further examined the acute toxicity of the Biotin-ss-CPT on nude mice. As shown in Figure 5(A), the heart, liver, spleen, lungs, and kidneys witnessed no obvious damage after treatment with Biotin-cc-CPT and Biotin-ss-CPT. However, it is worth noting that injection of equivalent dosages of CPT gave rise to liver damage and renal injury (denoted by arrows). This symbolized Biotin-ss-CPT have no apparent side effects on mice in general, while CPT still had.

Besides, the blood biochemistry test show that treatment with Biotin-ss-CPT effectively alleviated the toxicity of free CPT such as cardiotoxicity, hepatotoxicity, and nephrotoxicity in tumor-bearing nude mice (Figure 5(B)), as reflected by the liver function indicators (ALB, albumin; AST, aspartate aminotransferase; TP, total protein; GLB, globulin), kidney function indicator (BUN, blood urea nitrogen) and myocardial enzyme indicator (LDH, lactate dehydrogenase).

The quantitative determination of drug concentration in tumor region and the normal tissue were also detected (Figure 5(C)). The drug accumulation in heart, spleen, and lung for Biotin-cc-CPT, Biotin-ss-CPT, and CPT were close to each other. While it was noted that the drug concentration for Biotin-ss-CPT group in liver and kidney were obviously lower than CPT group, meanwhile, the concentration of former in tumor site were higher than that of the later. This symbolized that the biodistribution prodrug effectively decreased the accumulation in normal tissue and enhanced the accumulation in tumor site. In the light of the systematic toxicity results, the synthetic prodrug Biotin-ss-CPT could flexibly recognize and accumulate in the tumor site and apparently alleviate the side effects of CPT.

In addition, the tumor GSH levels and GPXs activity were also measured, the changes of them were determined in mice tumor after drug treatment for 24 h. The results show that GSH levels in tumor of Biotin-cc-CPT-treated mice decreased slightly in comparison with control groups. While GSH levels in the tumor of Biotin-ss-CPT-treated mice decline dramatically to 16%, even more obvious than that of CPT (24%) (Figure 5(D)). Similarly, treated mice with Biotin-ss-CPT resulted in a sharp decline of tumor GPXs activity (22%) as reflected in Figure 5(E), lower than CPT did (29%), demonstrating that the GPXs activity were remarkably inhibited by prodrug Biotin-ss-CPT. In the light of these data, Biotin-ss-CPT effectively perturb GSH/GPXs system in the tumor site, decreased the accumulation in normal tissue, and enhanced the accumulation in tumor site.

## 4. Discussion

In this study, we presented a biotin-conjugated prodrug Biotin-ss-CPT that was composed of biotin, S–S bond linker, and CPT. The anticancer activity of Biotin-ss-CPT was far superior to the counterpart Biotin-cc-CPT and close to the parent drug CPT. This is because Biotin-ss-CPT was able to react with intracellular thiol to liberate CPT and Biotin-cc-CPT could not. Moreover, Biotin-ss-CPT exerted higher cellular uptake and higher selectivity between cancer and normal cells than CPT. *In vitro* cytotoxicity test revealed that Biotin-ss-CPT effectively suppressed the migration of MGC803 cells and induce apoptosis in MGC803 cells involving in mitochondrial fission, alterations in Δ*Ψm*, generation of ROS and activation of caspases, as well as reducing GSH levels and inhibiting GPXs activity.

Although the potent anticancer activity of CPT is attractive and fascinating, the notorious side effects of CPT has become the main obstacle to further applications. *In vivo* investigation showed that Biotin-ss-CPT exerted a similar effect to CPT on the antiproliferation and growth of xenograft MGC803 cancer which confirmed by MR T2 weighted image and ADCs. However, toxicity evaluation showed that Biotin-ss-CPT displayed much higher selectivity than CPT that CPT caused noticeable liver damage and renal injury and weight loss while Biotin-ss-CPT did not. In summary, the synthetic prodrug Biotin-ss-CPT can be recognized specifically and selectively and taken up preferentially by cancers cells, followed by localization and accumulation effectively in the tumor site, then liberated CPT by the biological response to realize precise cancer therapy, which underlying further clinical applications.
